# Dissimilarity-driven behavior and cooperation in the spatial public goods game

**DOI:** 10.1038/s41598-019-44184-5

**Published:** 2019-05-21

**Authors:** Yinhai Fang, Tina P. Benko, Matjaž Perc, Haiyan Xu

**Affiliations:** 1 College of Economics and Management, Nanjing University of Aeronautics and Astronautics, Nanjing, 211106 China; 2 Faculty of Natural Sciences and Mathematics, University of Maribor, Koroška cesta 160, SI-2000 Maribor, Slovenia; 3 CAMTP – Center for Applied Mathematics and Theoretical Physics, University of Maribor, Mladinska 3, SI-2000 Maribor, Slovenia; 4 Complexity Science Hub Vienna, Josefstädterstraße 39, A-1080 Vienna, Austria

**Keywords:** Statistical physics, Sustainability, Evolutionary theory

## Abstract

In this paper, we explore the impact of four different types of dissimilarity-driven behavior on the evolution of cooperation in the spatial public goods game. While it is commonly assumed that individuals adapt their strategy by imitating one of their more successful neighbors, in reality only very few will be awarded the highest payoffs. Many have equity or equality preferences, and they have to make do with an average or even with a low payoff. To account for this, we divide the population into two categories. One consists of payoff-driven players, while the other consists of dissimilarity-driven players. The later imitate the minority strategy in their group based on four different dissimilarity-driven behaviors. The rule that most effectively promotes cooperation, and this regardless of the multiplication factor of the public goods game, is when individuals adopt the minority strategy only when their payoff is better than that of their neighbors. If the dissimilarity-driven players adopt the minority strategy regardless of the payoffs of others, or if their payoff is the same, the population typically evolves towards a neutral state where cooperators and defectors are equally common. This may be beneficial when the multiplication factor is low, when defectors would otherwise dominate. However, if the dissimilarity-driven players adopt the minority strategy only when their payoff is worse than that of their neighbors, then cooperation is not promoted at all in comparison to the baseline case in the absence of dissimilarity-driven behavior. We explore the pattern formation behind these results, and we discuss their wider implications for the better understanding of cooperative behavior in social groups.

## Introduction

In the last two decades, evolutionary game theory has strongly developed into a popular research area with a list of applications spanning from biology to social and economic systems^1–5^. Usually, interactions in the human society and economic systems cannot best described by well-mixed models but rather by models using networks^6–8^. In particular, spatial public goods game as one of the most popular evolutionary game model has become a powerful tool and provided many insights into the reasons for the emergence of cooperation affected by complex interactions and dynamics^9–17^. A famous case is the social dilemma^18^, where the interests of individuals are at odds with what is best for the community as a whole. Seeking the maximization of payoff is a surprisingly appropriate description of selfish players and most quantified by a scalar payoff value usually account for the vast majority of public goods game research^19–22^. In general, current research has highlighted two directions boosting cooperation in public goods game. One is the influence of network reciprocity on the evolution of cooperation including lattices^23^, scale-free graphs^24–27^, small-world graphs^28–30^ and multilayer networks^31–35^. The other is the impact of different decision mechanisms on the evolution of cooperation, including volunteering^36^, memory^15,37^, preference selection^38,39^, movement^40,41^, and aspiration^42–44^, to name just some. Heterogeneous update mechanisms in evolutionary games, in particular the mixing of innovative and imitative dynamics^45^, have recently also been considered in social dilemmas, with the conclusion being that this can be negative for the evolution of cooperation, especially near phase transition points.

Recently, the consideration of conformity has also attracted considerable attention as a possible mechanism to promote cooperation in the public goods game^46–51^. The gist of the idea is that conformity-driven individuals are those that simply adopt the most common strategy among their neighbors at any given time, whilst their respect for the expected payoff is merely of secondary importance. It has been shown that this can give rise to fascinating evolutionary dynamics, including such were cooperation is due to an enhanced coordination among the cooperators. It was also shown that cooperators focusing on payoffs and cooperators focusing on conformity can form an alliance against defectors, which is more effective than the efforts of either strategy individually^51^. Nevertheless, the importance of conformity must also be seen in the light of the general pursuit of dissimilarity and differentiation from the masses, which is frequently present in social interactions. In general, many individuals like to stand out, and indeed eschew conformity as a way of achieving precisely that. Social phenomena related to this are the emergence of different subcultures and alternative movements, which thus indicate that conformity is oftentimes neither the dominant nor the main motivator for a particular type of behavior.

Research has in fact shown that dissimilarity, as an antidote to conformity, has many different impacts on social activity of individuals and groups^52–54^. Behavior that is motivated by dissimilarity or by seeking variety has been studied particularly thoroughly for consumer behavior, where it is often associated with overthrowing certain fashion trends and gradually replacing them with new ones^55,56^. Similarly, in studies of organization, dissimilarity-driven behavior is frequently associated with trouble-making or reform, where such so-called mavericks often lead to innovation, provided their views are at least moderately appealing to the main stream^57^. Research has also shown that happiness is elevated and can be preserved over longer periods of time if variation in behavior or emotional stimuli is provided^58^. In groups, individuals with positions opposite to the majority are often seen as confident and innovative, which can in turn exert a remarkable influence on the majority via the so-called minority influence^59–62^. In fact, the convergent-divergent theory posits that majority ideas are conducive to convergence, while minority ideas are conducive to divergence^63^. Obviously, the later can be either towards positive, but also towards negative social change.

Using the above-described research as motivation, we here study how dissimilarity-driven players, i.e., players that strive simply to be different from others in the group regardless of their expected payoffs, impact the evolution of cooperation. As the basis model, we use the public goods game on the square lattice^2^, where we then consider four different player types that aspire to some form of dissimilarity. Intuitively, it is difficult to fathom the effects of dissimilarity-driven players. One may expect that the introduction of dissimilarity-driven players will make the population more neutral in terms of strategy representation rather than foster either cooperation or defection, but interestingly, as we will show, this is not always the case. In fact, we will show that dissimilarity-driven behavior can promote cooperation, especially so when individuals adopt the minority strategy only when their payoff is better than that of their neighbors. Other types of considered dissimilarity-driven behavior will tend to be less efficient in promoting cooperation, and indeed tend to neutralize the population. This is sometimes beneficial if compared to the baseline case with no dissimilarity-driven players, in particular if the multiplication factor of the public goods game is low, and sometimes not. We will also show that if the dissimilarity-driven players adopt the minority strategy only when their payoff is worse than that of their neighbors, then cooperation is not promoted at all in comparison to the baseline case. We will conclude with a discussion of the importance of these results for the better understanding of cooperative behavior in social groups, and we will also briefly outline possible applications where large-scale cooperation is particularly important.

## Results

**Figure 1:**
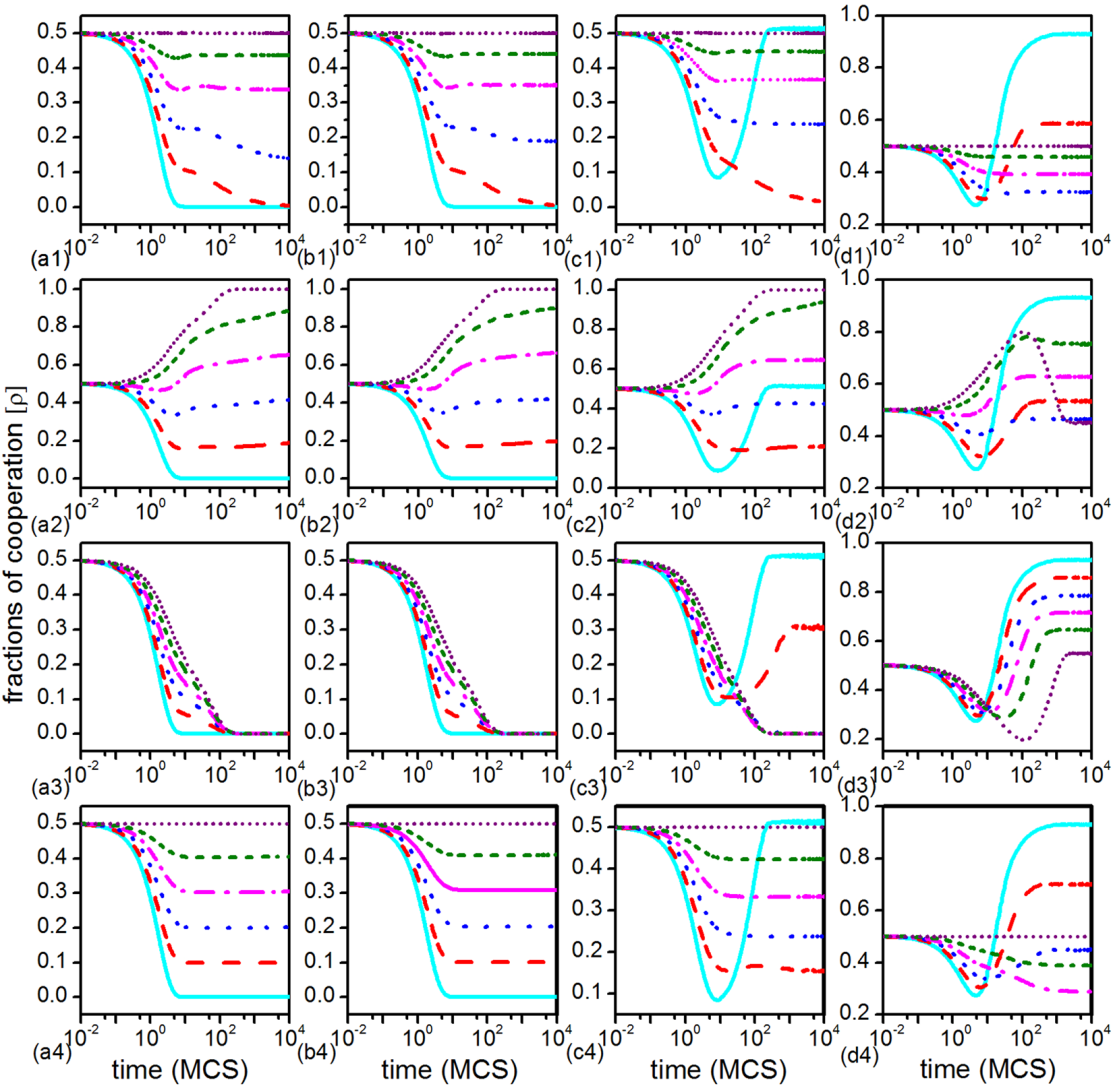
The evolution of the cooperator frequency (*ρ*) in dependence on the Monte Carlo time, as affected by four different dissimilarity-driven behaviors. From left to right (panels a–d) the results were obtained for synergy factor values *R* = 2, 3, 4 and 5, respectively, while from the top to bottom (panels 1–4) we consider the four different behaviors of type-A, type-B, type-C and type-D, respectively. Different lines in each panel were obtained for *β* = 0 (cyan solid line), *β* = 0.2 (red dashed line), *β* = 0.4 (blue dotted line), *β* = 0.6 (magenta dash-dotted line), *β* = 0.8 (olive short-dashed line) and *β* = 1 (purple short-dotted line). Other parameter values are *k*_*h*_ = 2, *N* = 4 × 10^4^, and *K* = 0.5. To further improve accuracy, the final results were averaged over 20 independent realizations, including the random initial strategy distributions and maverick distributions, for each set of parameter values.

In order to increase the understanding of the impact of dissimilarity-driven rule on public goods game, we plot the evolution of cooperators (*ρ*) with four types of behavior combining different proportion (*β*) for different synergy factor (*R*) in Fig. 1. The characteristic of evolution of cooperators varies greatly under the guide of different behaviors. For the type-A case (the individual with this behavior cares about nothing but the dissimilarity of the strategies among its neighbors and it adapts its strategy only according to the dissimilarity-driven rule) (Fig. 1(a(1)–d(1))) as *R* increases from 2 to 5 respectively, we can clearly see that, when individuals only driven by dissimilarity and never think about the payoff, the system tends to be neutral state as the fraction of dissimilarity-driven player increase. The dissimilarity-driven has a positive role in promoting the cooperation and avoids the system fall into an all-Ds state when *R* is small (e.g. *R* = 2, 3). It turns to be an negative effect when *R* is relatively large and just a small fraction of type-A behavior will hinder cooperation (e.g. *R* = 5).

However, the evolution of cooperation varies differently when players are guided by type-B behavior (who will adopt the minority strategy according to dissimilarity-driven rule only when its payoff is better than its neighbors), as presented in (Fig. 1(a(2)–d(2))). We can clearly see that type-B behavior sustains higher levels of cooperation even for small values of synergy factor (e.g. *R* = 2, 3). Especially, the system can tend to be all-Cs when *β* = 1 when *R* = 2, 3, 4. But it is important to point out that the positive effect of type-B behavior has been inhibited gradually as the increase of *R*. For example, low level of type-B behavior among the population has a positive effect on the cooperation at the beginning and then turns to negative effect while high level of type-B behavior has always positive effect during the whole evolution when *R* = 4. As *R* increase to 5, the existence of type-B behavior has an obvious negative effect on cooperation no matter *β* is high or low.

Fig. 1(a(3)–d(3)) show the impact of type-C behavior (individuals update strategy according to the dissimilarity-driven rule only when their payoff is less than neighbors) on cooperation. It can be seen that the existence of type-C behavior can slow down the decay of cooperation, but in the end the whole system will still converge to the all-Ds state when *R* is small (e.g. *R* = 2, 3). Fig. 1(c(3)–d(3)) show that there are two opposite effects in the evolution process of cooperation. type-C has a positive effect on the cooperation at the beginning and then on the contrary.

From Fig. 1(a(4)–d(4)), one can see that the characteristics of evolution guided by type-D behavior (individuals change their strategy guided by dissimilarity-driven rule when they their payoff is equal with the chosen neighbor) is similar to type-A behavior, and the system tend to be neutral state with the increase of dissimilarity-driven individuals among the population. In general, it is clear that type-B behavior of dissimilarity-driven rule is most beneficial for the promotion of cooperation. Moreover, type-A and type-D behavior can cause the system to approach a neutral state with the increase of *β*. This is very meaningful when *R* is small (e.g. *R* = 2, 3), which can prevent the system from evolving to an all-Ds state.

**Figure 2:**
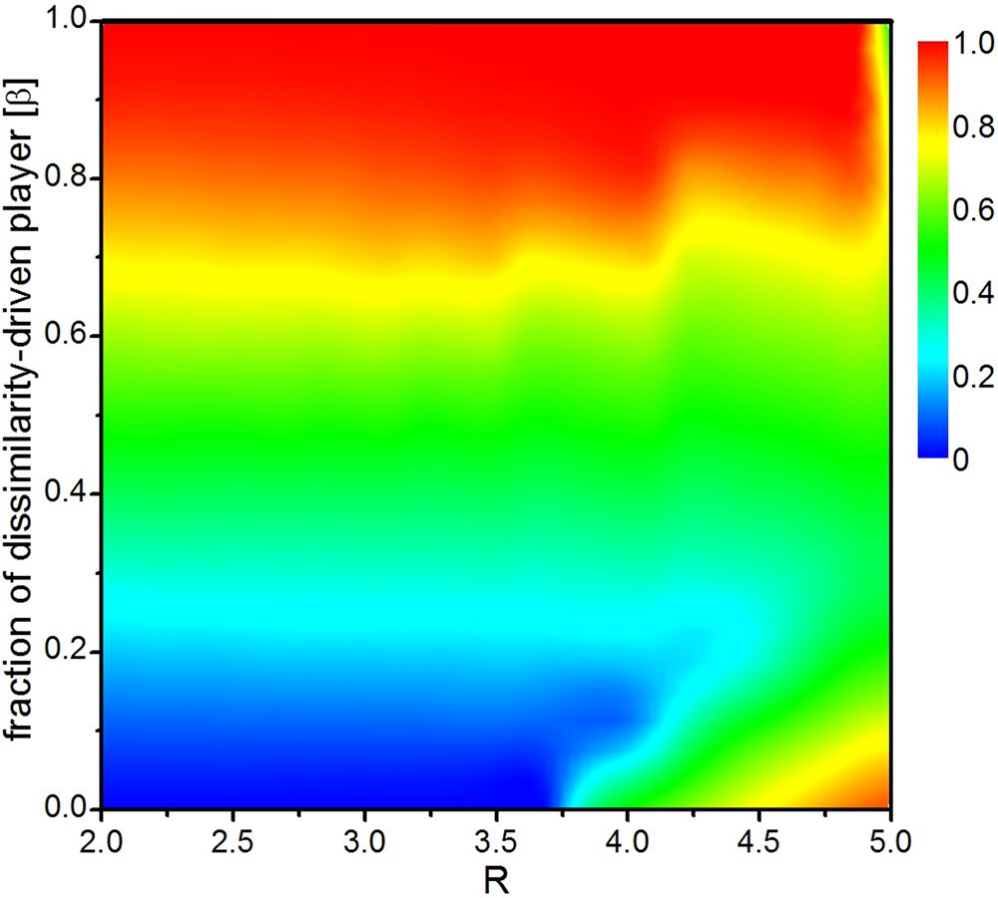
Cooperation diagram on varying *R* and *β* in a population with type-B behavior of dissimilarity-driven rule. *N* = 4 × 10^4^, *k*_*h*_ = 2, *K* = 0.5, *R* in the range ∈ [2.0, 5.0], *β* in the range ∈ [0.0, 1.0]. Results are averaged over the last 5000 steps of 25000 MCS and have been computed using 31 × 11 parameter values.

The positive effect of type-B is influenced by the increase of *R* as shown in Fig. 1. In order to investigate the combined effect of *β* and *R* on cooperation, we present the colour map encoding the fraction of cooperation on the plane *β*–*R* (i.e., density of type-B dissimilarity-driven behavior vs synergy factor) in Fig. 2. At a first glance, cooperators survive only if *R* > 3.74 in the absence of dissimilarity-driven individuals, as also reported in the existing literature^64^. Then, we observe that as *β* increases, cooperation gradually increases when fix the value of *R*. For example, we fix *R* = 2.6, when *β* = 0, *ρ* = 0; *β* = 0.6, *ρ* = 0.7; *β* = 1, *ρ* = 1. However, there is significant difference between the fractions of cooperation comparing *R* < 3.74 and *R* > 3.74. In particular, the positive effect of type-B on cooperation is monotonous if *R* < 3.74 and then turns to be non-monotonous when *R* > 3.74. Another point to note is that the system can tend to be all-Cs if the value of *β* is large enough (e.g. *ρ* = 1 when *β* = 1 for *R* = 2, 3, 4). In general, it is obvious that the type-B behavior has a positive effect on the cooperation for a wide range of *R*. That is to say, the existence of type-B dissimilarity-driven players among the population enables the cooperators to survive, and a large *β* value could significantly promote cooperative behavior.

**Figure 3:**
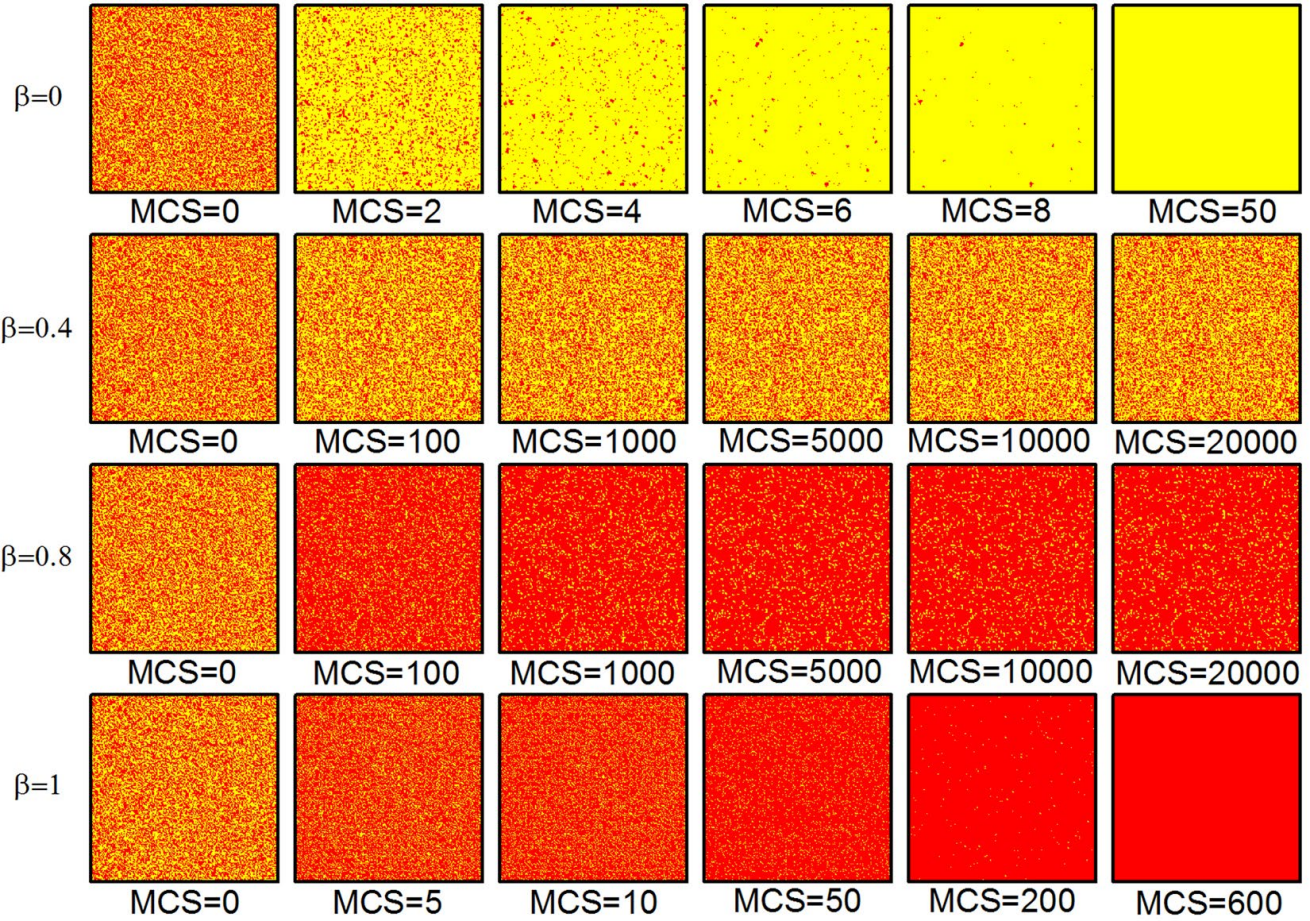
Snapshots of typical distributions of cooperators (red) and defectors (yellow) guide by the type-B behavior of dissimilarity-driven rule at different Monte Carlo time when *R* is 3. From the top to the bottom, *β* = 0, *β* = 0.4, *β* = 0.8 and *β* = 1, respectively, as well as all results are obtained for *k*_*h*_ = 2, *N* = 4 × 10^4^, *K* = 0.5.

To illustrate the reason of improvement of cooperation by introduction of the type-B dissimilarity-driven player, we study the evolution of the strategy pattern starting with the random initial states for different *β* at low synergy factor (i.e., *R* = 3) in Fig. 3. It shows a complete picture on the time evolution of cooperators and defectors. For traditional case presented in the first level of Fig. 3, players are all payoff-driven individuals. We can clearly see that defectors spread very efficiently and have a good change to form large clusters rapidly without the type-B behavior. Eventually, the system tends to be all-Ds and cooperators are completely wiped out in a short time. However, when the type-B dissimilarity-driven player is introduced into the group, such as *β* = 0.4 and 0.8, we can find that compact clusters of cooperators formed and scattered among the population, which have a good change to invade defectors and the defectors cannot form clusters. Of course, the cooperators are always in a dominant position when *β* is large. Thus, the larger the fraction of type-B behavior players in the system, the higher fraction of cooperators among the population. In particular, defectors extinct at the end less than 600 MCS when *β* = 1. Therefore, the type-B behavior in a group has played a very important role in the promotion of cooperative behavior when synergy factor is not very high.

**Figure 4:**
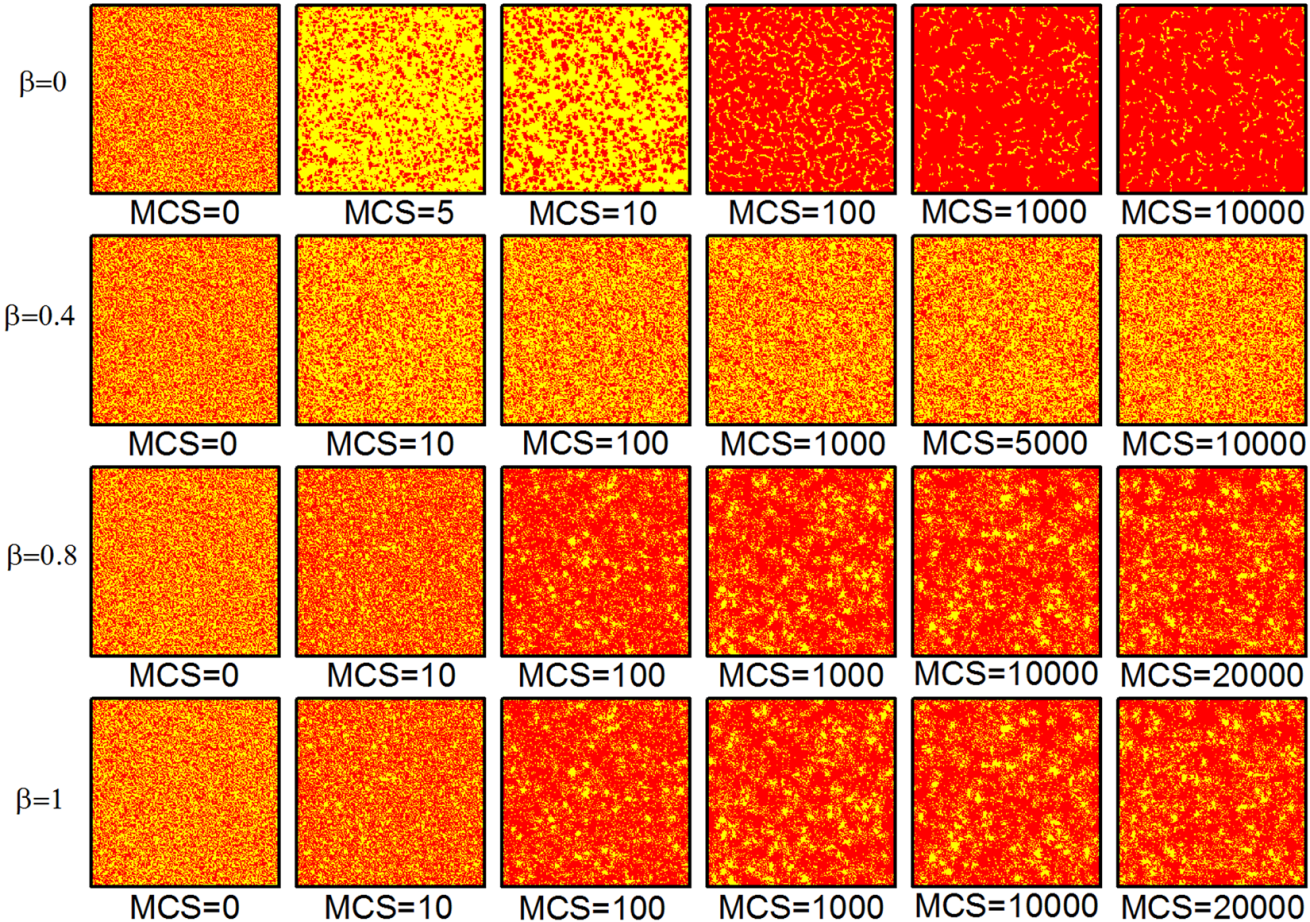
Characteristic snapshots of cooperation (red) and defection (yellow) strategies with the effect of type-B behavior of dissimilarity-driven rule when the value of *R* is 5. From top to the bottom the value of *β* equals to 0, 0.4, 0.8 and 1, respectively for different MCS steps, as well as all results are obtained for *k*_*h*_ = 2, *N* = 4 × 10^4^, *K* = 0.5.

To investigate the reason why type-B behavior loses the function of promoting cooperation in Figs 1 and 2 when *R* = 5, we show a series of characteristic strategy distributions that describe the time evolution of the system from a random initial state in Fig. 4. The system enters a high cooperative stable state more quickly without the type-B behavior (*β* = 0). Notably, we observe that type-B behavior hinders the formation of large cluster of cooperators when *β* is smaller than 0.5 (e.g. *β* = 0.4), obviously, which has a negative effect on the cooperation. On the other hand, it is worth noting that, for *β* is larger than 0.5, large cluster of cooperators can spring up during the evolution. But it is not the same case as Fig. 3, small clusters of defectors have also been formed. This is the reason why type-B behavior lose its positive effect on cooperation.

**Figure 5:**
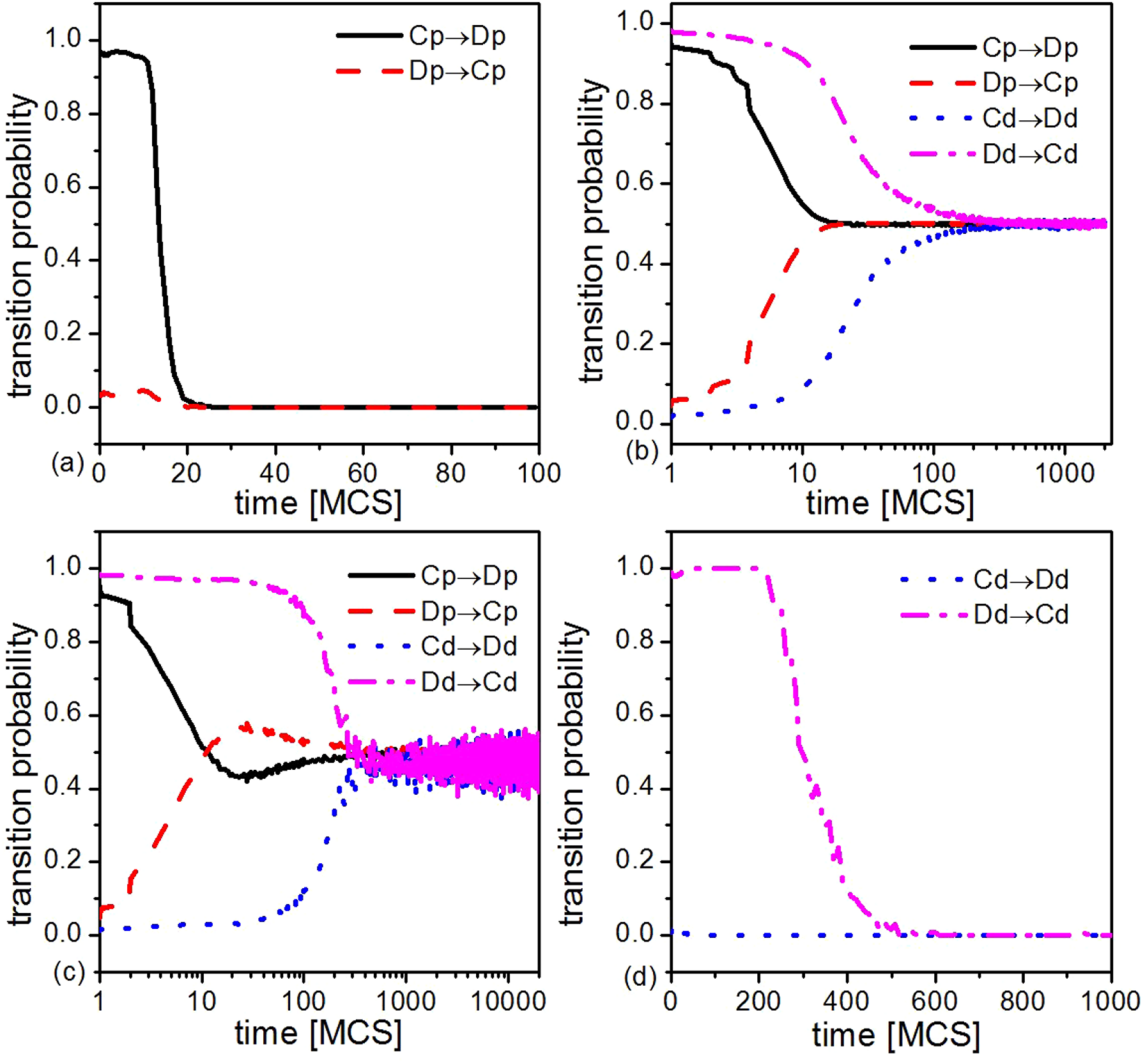
The transition probability of strategies as a function of MCS steps for *β* equals to 0, 0.4, 0.8 and 1 from (**a**–**d**), respectively. All the results are obtained for *R* = 3, *k*_*h*_ = 2, *N* = 4 × 10^4^, *K* = 0.5. To improve accuracy, the final results are averaged over 500 independent realizations, including the random initial strategy distributions and dissimilarity-driven player distributions for each set of parameter values.

Finally, it is worthwhile for us to study how strategies are transformed, which is necessary for us to understand the direct reason why cooperation emerges after the introduction of dissimilarity-driven players. Fig. 5 presents the transition probability of strategies as a function of MCS step for *β* equals to 0, 0.4, 0.8 and 1 from (a)–(d), respectively. Here, *C*_*p*_ → *D*_*p*_ (*D*_*p*_ → *C*_*p*_) and *C*_*d*_ → *D*_*d*_ (*D*_*d*_ → *C*_*d*_) describe the transition probabilities of cooperators (defectors) changing into defectors (cooperators) among payoff-driven players and dissimilarity-driven players (type-B behavior), respectively. When *β* = 0 (Fig. 5(a)), all players are payoff-driven, a high fraction of cooperators changing into defectors, and only a small fraction of defectors changing into cooperators at the beginning of evolution and then the system reaches a steady state (all-Ds) quickly. As the value of *β* increases to 0.4 and 0.8 (Fig. 5(b,c)), on the one hand, the transition probability of *D*_*p*_ → *C*_*p*_ gradually increases. Especially, it is larger than the transition probability of *C*_*p*_ → *D*_*p*_ during a period of evolution when *β* equals to 0.8. On the other hand, the transition probability of *D*_*d*_ → *C*_*d*_ is much larger than the transition probability of *C*_*d*_ → *D*_*d*_ during the beginning period of evolution and then get into a balanced state. When *β* = 1 (Fig. 5(d)), all individuals are dissimilarity-driven players (type-B behavior), the transition probability of *D*_*d*_ → *C*_*d*_ is close to 1 and then decrease gradually, which is much larger than the transition probability of *C*_*d*_ → *D*_*d*_ during the whole evolution. Thus, the introduction of type-B behavior of dissimilarity-driven players will lead to cooperators survive in the system.

## Discussion

Inspired by ample research on dissimilarity in social activity and its effects on consumer behavior, organization in groups, as well as happiness and minority influence^52–62^, we have here studied the impact of dissimilarity-driven behavior on cooperation in the public goods game on a square lattice. Existing research has paid plenty of attention on conformity behavior. However, with the development of economic globalization, people’s view is gradually pursuing individualization and differentiation. Thus, we have aimed to understand the impact of dissimilarity-driven behavior in the public goods game. In particular, the individuals driven by dissimilarity are those that tend to adopt the strategy of the minority instead of imitating the richest neighbors driven by payoff. To find out the impact of dissimilarity-driven individuals on cooperation and the reasons why the effects occur, we have considered four different types of dissimilarity-driven behavior, namely players adopting the minority strategy only when their payoff is better than that of their neighbors, players adopting the minority strategy only when their payoff is lower than that of their neighbors, players adopting the minority strategy only when their payoff is equal to that of their neighbors, and players adopting the minority strategy regardless of the payoffs of others.

Through extensive simulations, we have shown that the type-B dissimilarity-driven behavior, when individuals adopt the minority strategy according to dissimilarity-driven rule only when their payoffs are better than their neighbors, plays an important role in the promotion of cooperators, such that the the greater the value of *β*, the higher the level of cooperators among the population. It is important to note that the positive effect is monotonous with the increase of *β* when *R* < 3.74 and then becomes non-monotonous when *R* > 3.74, which means that the positive effect of type-B behavior is gradually weakening. Especially, the system can tend to be all-Cs when *β* is large enough among a wide range of *R*. But, the all-Cs state will never occur when *R* is large enough (e.g. *R* = 5). Overall, our research indicates that type-B behavior can promote cooperation in the public goods game with a wide range of *R*. Type-A (individual with this behavior cares about nothing but the dissimilarity of the strategies among its neighbors and it adapts its strategy only according to the dissimilarity-driven rule) and type-D (individuals change their strategy guided by dissimilarity-driven rule when they have the equal payoff with the chosen neighbor) behavior can cause the system to approach a neutral state with the increase of *β*. This is potentially beneficial when *R* is small (e.g. *R* = 2, 3), which can prevent the system from evolving to an all-Ds state. Lastly, we note that type-C behavior (individuals update strategy according to the dissimilarity-driven rule only when their payoff is less than neighbors) has a limited positive effect on cooperation and it can only delay the system evolve into an all-Ds state and then this limited positive effect completely transformed into inhibition as *R* increases.

We hope that these results can improve our understanding of cooperation in groups, in particular where large-scale cooperation is particularly important, such as with vaccination^65–67^, the responsible use of antibiotics^68^, or for mitigating climate change and adverse environmental effect of overexploitation of natural resources^69^.

## Methods

We use two variations of public goods game, which we have referred to as the ‘payoff-driven’ and ‘dissimilarity-driven’ models, concertized on a regular square lattice of size *L*^2^ under periodic boundary conditions. Initially, each player on site *x* is assigned as a cooperator (*s*_*x*_ = *C*) or defector (*s*_*x*_ = *D*) with equal probability and designated as dissimilarity-driven or payoff-driven at the same time. Individuals play the game with their four neighbors. Thereby, each individual belongs to five different groups (i.e., it is the focal individual of a Moore neighborhood and a member of the Moore neighborhood of its four nearest neighbors) (see^2^ for a review).

Using standard parametrization, each cooperator donates 1 to the public goods while defector donates nothing. The sum of all donations is multiplied by the factor (*R* > 1), reflecting the synergy factor of cooperation, and the resulting amount is subsequently equally shared among the *k* + 1 interacting individuals regardless of their strategies. Denoting the number of *C*_*s*_ and *D*_*s*_ among the four interacting partners by *N*_*C*_ and *N*_*D*_ respectively, each cooperator gets

1 $${P}_{C}=R\times ({N}_{C}+1)/(k+1)-1$$

while a defector receives

2 $${P}_{D}=R\times {N}_{C}/(k+1)$$

After the two kinds of players on the *L*^2^ square lattice distributed uniformly at random with different fractions, random sequential update steps of traditional payoff-driven is presented as below. A randomly selected player *x* plays the public goods game with the *k* interacting players of a group *g*, and obtains an accumulative payoff ${P}_{x}=\sum _{x\in g}\,{P}_{{s}_{x}}$. Then, one of the four nearest neighbors of player *x* is chosen randomly, and its location is denoted by *y*. Player *y* also acquires its accumulative payoff *P*_*y*_ identically as previously player *x*. Player *x* imitates the strategy of player *y* with the probability

3 $$q=1/\{1+\exp [({P}_{x}-{P}_{y})/K]\}$$

Here, *K* quantifies the uncertainty of strategy updating process^70^. In consistent with previous works, we select *K* = 0.5 ensuring that strategies of better-payoff players are readily adopted by their neighbors, although adopting the strategy of a player that performs worse is also possible. Such errors in adoption process can be attributed to imperfect information, mistakes in the evaluation of the opponent, and some other similar factors.

In order to introduce dissimilarity-rule in the public goods game, we consider four different behaviors of dissimilarity-driven players. The first is the absolute dissimilarity-driven behavior. The individual with this behavior cares about nothing but the dissimilarity of the strategies among its neighbors and it adapts its strategy only according to the dissimilarity-driven rule. We call this kind of behavior as type-A. The second kind of individuals who will adopt the minority strategy according to dissimilarity-driven rule only when its payoff is better than its neighbors. This kind of behavior is set as type-B. The third kind of individuals update strategy according to the dissimilarity-driven rule only when their payoff is less than neighbors. This is set as the type-C behavior. The last kind of individuals change their strategy guided by dissimilarity-driven rule when they have the equal payoff with the chosen neighbor. We call this kind of behavior as type-D. In detail, each dissimilarity-driven player simply prefers to adopt the strategy that is least common within its interaction range. Thus equation (3) will no longer be applied. Instead, if player is dissimilarity-driven, the probability of strategy adaption determined as below

4 $${\rm{\Gamma }}({N}_{{s}_{x}}-{k}_{h})=1/\{1+\exp [({k}_{h}-{N}_{{s}_{x}})/K]\}$$

Here, ${N}_{{s}_{x}}$ is the number of players adopting strategy *S*_*x*_ within the interaction range of player *x*. *k*_*h*_ is one half of the degree of player *x*. It is worth pointing out that the application of equation (4) results in the dissimilarity-driven adopting, with a very high probability, whichever strategy (either *C* or *D*) is at the time the least common in its neighborhood. However, it is still possible, yet very unlikely that a dissimilarity-driver will adopt the strategy that is in the majority. Nevertheless, if the number of cooperators and defectors in the neighborhood is equal, the dissimilarity-driven player will change its strategy with probability 0.5 (see also^71,72^ for reviews of similar adaptations of the evolutionary dynamics).

We simulate the model in accordance with the standard Monte Carlo simulation procedure. Initially, the cooperators and defectors are randomly distributed among the population with equal probability. Payoff-driven rule and conformity-driven rule are assigned to players with probabilities *β* and 1 − *β* among population respectively. Each full Monte Carlo step (MCS) consists of *N* (*L* × *L*) elementary steps described above, which are repeated successively, thus giving a chance to every player to alter its strategy once on average during a full Monte Carlo step. All simulation results are obtained on the square lattice that usually consisted of *N* = 4 × 10^4^ players.
